# Determination of thresholds of risk in women at average risk of breast cancer to personalize the organized screening program

**DOI:** 10.1038/s41598-021-98604-6

**Published:** 2021-09-27

**Authors:** Emmanuel Bonnet, Jean-Pierre Daures, Paul Landais

**Affiliations:** 1 Montpellier University, EA2415, Institut Universitaire de recherche clinique, 34093 Montpellier Cedex 5, France; 2 Languedoc Mutualité, Nouvelles Technologies, AESIO, Montpellier, France

**Keywords:** Cancer screening, Breast cancer, Statistics, Risk factors

## Abstract

In France, more than 10 million women at ”average” risk of breast cancer (BC), are included in the organized BC screening. Existing predictive models of BC risk are not adapted to the French population. Thus, we set up a new score in the French Hérault region and looked for subgroups at a graded level of risk in women at ”average” risk. We recruited a retrospective cohort of women, aged 50 to 60, who underwent the organized BC screening, and included 2241 non-cancer women and 527 who developed a BC during a 12-year follow-up period (2006-2018). The risk factors identified were high breast density (ACR BI-RADS grading)(B vs A: HR = 1.41, 95%CI [1.05; 1.9], p = 0.023; C vs A: HR = 1.65 [1.2; 2.27], p = 0.02 ; D vs A: HR = 2.11 [1.25;3.58], p = 0.006), a history of maternal breast cancer (HR = 1.61 [1.24; 2.09], p < 0.001), and socioeconomic difficulties (HR 1.23 [1.09; 1.55], p = 0.003). While early menopause (HR = 0.36 [0.13; 0.99], p = 0.003) and an age at menarche after 12 years (HR = 0.77 [0.63; 0.95], p = 0.047) were protective factors. We identified 3 groups at risk: lower, average, and higher, respectively. A low threshold was characterized at 1.9% of 12-year risk and a high threshold at 4.5% 12-year risk. Mean 12-year risks in the 3 groups of risk were 1.37%, 2.68%, and 5.84%, respectively. Thus, 12% of women presented a level of risk different from the average risk group, corresponding to 600,000 women involved in the French organized BC screening, enabling to propose a new strategy to personalize the national BC screening. On one hand, for women at lower risk, we proposed to reduce the frequency of mammograms and on the other hand, for women at higher risk, we suggested intensifying surveillance.

## Introduction

Breast cancer is the most common cancer in women and the leading cause of death from cancer in women in France^1^. The earlier breast cancer is detected, the greater the chances of recovery. For a breast cancer detected at an early stage, 5-year survival is 99%, and 27% for a metastatic cancer^2^. The difference in the detection stage also influences the cost of medical care^3^. While high risk women^4^ including women with a deleterious constitutional mutation predisposing to breast cancer, women for whom a surgical intervention with biopsy has shown an histological risk factor as well as women with a personal history of breast cancer receive an individualized monitoring, the majority of women aged from 50 to 74 are included in the nationwide organized breast cancer screening. In France, this screening consists of a mammography (2 views) every two years with a double reading of mammographic images, and targets a population of more than 10 million women^5^. Current participation is about 50% with a target of 65%. The number of women actually screened in France is therefore between 5 and 6.5 million.

In April 2017, the Ministry of Health published an action plan to renovate the organized breast cancer screening^6^ based on the recommendations of the National Cancer Institute and at the request of the women interviewed during the citizen consultation to determine the levels of risk for the population at ”average” risk^7^. This reorganization plan aimed to enable a more personalized organized screening, and encouraged research projects on tools and methods for assessing the level of risk, including scoring. The aim of our study was to explore whether, among the 10 million women at ”average-risk” targeted by the French organized screening, some women would be at lower-risk than average risk and others at higher risk. The aim of this personalized organized screening is to optimize the available resources to make screening as efficient as possible. As it was a request from the women interviewed during the citizen consultation, it could also make them more involved in organized breast cancer screening and increase the participation rate.

Several predictive models already exist, such as Gail^8^, BRCAPRO^9^, BODICEA^10^, Tyrer-Cuzick^11^, or PROCAS^12^ models. But they are not adapted to some European populations^13^ and most of them include BRCA1 and BRCA2 mutations. However, in France, women presenting with these mutations are not included in the organized nationwide screening and, as explained in PROCAS^12^, mutations in breast cancer genes such as BRCA1 and BRCA2 are too infrequent to affect risk prediction appreciably in the models for the general population. Moreover, it is difficult to estimate an individual risk precisely^14,15^.There are also some studies on European populations performed in England^12^. We, therefore, intended to set up a new risk model, including new variables, adapted to the French population, and enabling to explore whether in this average risk population it was possible to identify women at lower risk of breast cancer (or higher risk, respectively) in order to adapt the organized screening accordingly by proposing a different organized screening strategy depending on the level of risk identified.

## Material and methods

### Database

We used the database of the Center that coordinates breast cancer screening (CRCDC-OC) in the Hérault department, France. This database registers women with precise, verified, and consolidated information on the occurrence of breast cancer during the follow-up of their screening. The data are also consolidated by the Hérault tumor register which provides an exhaustive information on the occurrence of breast cancer, including interval cancers during the follow-up period. We enrolled women, aged 50 to 60 at inclusion, who experienced a 12-year breast cancer screening from 2006. We extracted a database of 33,858 women, including 805 women who developed a breast cancer in 3-12 years following inclusion. We did not include women who have been diagnosed with breast cancer during the first two years of follow-up to avoid including women who might have already developed a cancer prior to inclusion, but not yet diagnosed at inclusion. Data were recovered by a telephone survey. It was not possible to interview 33,000 women. Therefore, on one hand we selected all the women who developed a breast cancer during the follow-up period, and on the other hand, we randomly selected 5000 women among those who did not develop breast cancer using the *sample* function of the R software applied to the list of 32,962 unique identifiers.

### Power

We hypothesized a risk ratio $\ge 2$, with a ratio of 4 to 1 between women who did not develop breast cancer and those who did during the follow-up period, together with a power of 80%. It gave a required sample size of 543 women with breast cancer during the follow-up period and 2174 women without breast cancer.

### Study questionnaire

We looked for the risk factors for breast cancer in average risk women reported in the literature prior to creating the study questionnaire. We elaborated a text mining algorithm-assisted search (TEMAS) (available at shiny.temas-bonnet.site) to optimize the bibliographic search on very large volumes of scientific articles. We also included the variables used in Gail’s model relative to women at average risk. The study questionnaire is provided in Supplementary material 1.

### Data collection

**Figure 1:**
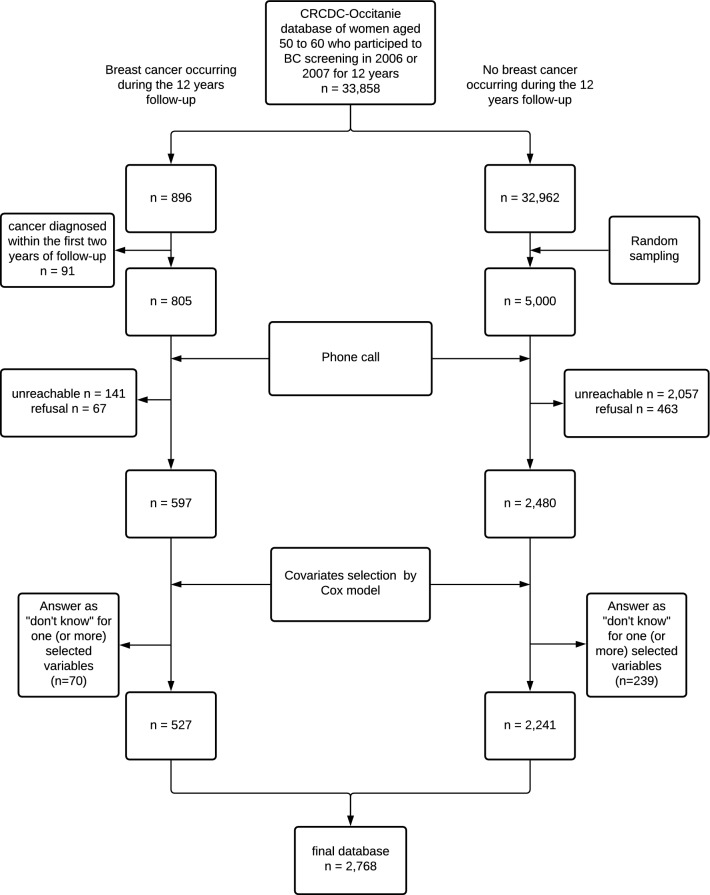
Flowchart of the retrospective cohort study.

Data were collected by telephone by two trained investigators. An explanatory leaflet (Supplementary material 3) was sent to all participants prior to the phone call and informed consent was obtained from all individual participants prior to the telephone interview. Responses to the study questionnaire were collected between November 2018 and May 2019. The inclusion flowchart is shown on Figure 1; 2,198 women were unreachable (mostly unassigned phone number, or reiterated unanswered calls) and 530 refused to participate (mostly alleging lack of time). Thus, this survey enabled collecting 3077 questionnaires, including 597 questionnaires from women who developed a breast cancer. The Case Report Form was developed with the ACCESS software. The analysis database was provided by the CRCDC-OC while having anonymized the data beforehand. This study complies the MR003 CNIL’s (National Commission for Informatics and Liberties) methodology (Declaration: 2200402v0). The study was also agreed by the ethics committee of the Personal Protection Committee (A0246039). All methods were performed in accordance with the relevant guidelines and regulations.

### Statistical analysis

Data management was performed on the fly. A data check was performed daily, especially on outliers and missing data. It enabled to call back women without delay if needed. Statistical analysis was carried out using R software^16^.

#### Descriptive analysis

We discretized the variables according to the literature. For duration of oral contraceptive^17^ we retained the classes 0, [1,5[, [5,10[, and $\ge$ 10. For the number of biopsies, we complied with Gail’s classes^8^ 0, 1, $\ge$ 2. Breast density was categorized in four classes according to the ACR BI-RADS (Breast Imaging-Reporting and Data System) Atlas^18^ : A: the breasts are almost entirely fatty, B: there are scattered areas of fibroglandular density, C: the breasts are heterogeneously dense, and D: the breasts are extremely dense. For the number of hours per week of physical activity we followed WHO recommendations^19^ : 0, [1-5[, and $\ge$ 5. Alcohol consumption (in glasses per week) was discretized as follows : 0, [1,10[, $\ge$ 10 according to Santé Publique France (French public health service) recommendations^20^. Age at menarche was divided as follow: < 12 and $\ge$ 12^21^. Age at first live birth was discretized in 3 classes^22^: nulliparous, $\le$ 24, and > 24. For number of children, we used the average number of children in the generation of interest^23^: 0, [1,3[, and $\ge$ 3. For the total duration of breastfeeding we followed WHO and UNICEF recommendations^24^: 0, [1,7[, and $\ge$ 7. For age at menopause, we used the definition of the national college of French gynecologists of early menopause^25^: < 41, $\ge$ 41. We created a socio-economic hardship binary variable based on the EPICES^26^ score. Women responded to the EPICES questionnaire, and we only kept the two covariates which were statistically significant in univariate models. The modalities of the binary covariate thus constructed were ”yes” if the interviewee presented financial hardship and / or was not going on vacation, and ”no” otherwise. We also studied overweight (i.e. BMI$\ge$25). We also studied detailed family history of breast cancer : mother, sister, daughter, and aunt. All quantitative variables were also tested as continuous variables.

#### Cox model

All variables with a P-value <0.2 in the univariate models were included in a global Cox model, then a selection with the stepwise Akaiké information criterion (AIC) was achieved (age was forced in the model) in order to obtain ${{\hat{\beta }}}$, the estimated coefficients of the Cox proportional hazards regression model. Schoenfeld residuals were computed to check the proportional hazards assumption.

Our goal was to define an individual score. Therefore, to estimate ${\hat{\Lambda }}_0$, the cumulative base-line hazard function, we used Breslow’s estimator^27^ defined below.

$$\begin{aligned} {\hat{\Lambda }}_0(t) = \sum _{i:T_i \leqslant t}\frac{d_i}{\displaystyle \sum _{j\in R(T_i)} \exp ({\hat{\beta }}^\prime Z_j)} \end{aligned}$$

with:

- $T_1<T_2<\dots <T_{12}$ distinct times to event
- $d_i$ the number of events detected at $T_i$
- $Z_i$ the value of the covariates for patient *i*
- $R(T_i)$ all individuals still at risk at time $T_i^-$ (just before $T_i$)
- ${{\hat{\beta }}}$ the estimated coefficients of the Cox proportional hazards regression model

It enabled to estimate a risk function *F* for each individual as a function of her covariates :

$$\begin{aligned} {\hat{F}}(t|Z) = 1-{\hat{S}}(t|Z) = 1- \exp \left( -{\hat{\Lambda }}_0(t) \, \exp ({\hat{\beta }}^\prime Z) \right) \end{aligned}$$

#### Bootstrap

As we performed a random sampling among women without breast cancer, in our database, the distribution between women who developed cancer and the others was no longer representative of the starting population. To remedy this, we performed bootstrap samples. Bootstrap is a resampling method that uses random sampling with replacement. This method allowed to avoid skewing cancer rates compared to the population of interest without introducing bias. We computed 100,000 bootstrap databases using stratified random sampling with replacement among women for whom we obtained answers to all the covariates of the final Cox model, in order to obtain a database close to the initial CRCDC database (i.e. 33,858 women, including 896 who developed a breast cancer within the 12 years of follow-up).

In these databases, mean risk was identical to the one of the CRCDC database. Then, we estimated, for each woman, $\Lambda _0(t)$, and calculated *F*(12|*Z*) to obtain a risk score of developing a breast cancer during the follow-up period.

#### Thresholds and likelihood ratios

In France, the number of women actually screened is between 5 and 6.5 million. Among these women, we hypothesized that 10% of them might have a risk different from the “average” risk of breast cancer, either lower or higher. This average 12-year risk was 2.6% in the organized screening population. From a clinical standpoint, we looked for women that might have a risk two times lower than this “average” risk. This group was considered as at lower-risk. We also looked for women that might have a risk two times higher than this “average” risk. This group was considered as at higher-risk. We then searched for the corresponding thresholds. We looked for $s_1$, the higher risk threshold, and $s_2$, the lower risk threshold, such as:

$$\begin{aligned}&\frac{\mathbb {P}(M|R>s)}{\mathbb {P}(M|R<s)}\ge 2 \; \;\; \;\; \; \forall s\ge s_1\\ and \\&\frac{\mathbb {P}(M|R<s)}{\mathbb {P}(M|R>s)}\le 0.5 \; \;\; \;\; \; \forall s\le s_2 \end{aligned}$$

where:

- M represents the event ”developing a breast cancer during the 12 years follow-up”;
- R represents the risk as determined by the score.

This method then enabled to delineate 3 groups according to the level of risk: lower risk, average risk, and higher risk.

Given that, for each threshold *s*, the likelihood ratios (LR) either positive (+) or negative (-) were as follows:

$$\begin{aligned} {\text{LR}} +(s) =\frac{{\text{sensitivity}}\, (s)}{1-{\text{sensitivity}}\, (s)} \;\;\;\;\;\;\;\;\;\;\;\;\;\;\; {\text{LR}} -(s) =\frac{1-{\text{sensitivity}}\, (s)}{{\text{sensitivity}}\, (s)} \end{aligned}$$

We plotted the curves LR+(s) and LR-(s) curves and looked for $s_1$ and $s_2$ according to the calculations available in Supplementary material 2.

Women with a risk score below the ”low threshold” will be considered at lower risk of breast cancer, women with a risk level between ”low threshold” and ”high threshold” will be considered at average risk and finally, women with a risk score above the ”high threshold” will be considered at higher risk of breast cancer in this population of women at average risk.

### Ethics approval

We obtained the authorization No.: 2200402v0 from the CNIL’s (National Commission for Informatics and Liberties). We also received the agreement (No.: A0246039) of the ethics committee of the CPP (Personal Protection Committee). All methods were performed in accordance with the relevant guidelines and regulations.

### Consent to participate

An explanatory leaflet (Supplementary material 3) was sent to all participants prior to the phone call and informed consent was obtained from all individual participants prior to the telephone interview.

### Consent for publication

Not applicable.

## Results

### Personalized organized breast cancer screening retrospective cohort: descriptive analysis

Descriptive analysis result is presented in Table 1. The significant risk factors in the univariate analysis were the number of biopsies ($p=0.042$), breast density ($p=0.012$), total duration of breastfeeding ($p=0.026$), financial difficulties and/or not going on vacation ($p=0.002$), mother history of breast cancer ($p=0.001$), and aunt history of breast cancer ($p=0.029$).

**Table 1:** Descriptive analysis.

Breast cancer		No n (%)	Yes n (%)	P-value
Age (years)	[49,53[	626 (25.2)	156 (26.1)	0.164
	[53,57[	915 (36.9)	196 (32.8)	
	[57,60]	939 (37.9)	245 (41.0)	
Oral contraceptive use	No	605 (24.5)	165 (27.7)	0.111
	Yes	1867 (75.5)	430 (72.3)	
Contraceptive pill duration of use (years)	0	605 (24.8)	165 (28.5)	0.156
	[1,5[	520 (21.3)	127 (21.9)	
	[5,10[	327 (13.4)	80 (13.8)	
	$\ge$ 10	986 (40.4)	207 (35.8)	
Number of breast biopsies	0	2182 (88.8)	506 (86.2)	0.042
	1	231 (9.4)	61 (10.4)	
	$\ge$ 2	45 (1.8)	20 (3.4)	
Breast density	A	338 (13.6)	60 (10.1)	0.012
(BI-RADS classification)	B	1483 (59.8)	348 (58.3)	
	C	604 (24.4)	168 (28.1)	
	D	55 (2.2)	21 (3.5)	
Physical activity (hours/week)	0	972 (39.3)	225 (37.7)	0.093
	[1,5[	1185 (47.9)	275 (46.1)	
	$\ge$ 5	318 (12.8)	97 (16.2)	
Alcohol consumption (glasses/week)	0	1329 (53.6)	344 (57.7)	0.16
	[1,10[	920 (37.1)	197 (33.1)	
	$\ge$ 10	229 (9.2)	55 (9.2)	
Age at menarche (years)	< 12	483 (20.1)	133 (23.1)	0.121
	$\ge$ 12	1919 (79.9)	442 (76.9)	
Age at first live birth (years)	$\le$ 24	1361 (55.0)	334 (56.4)	0.226
	>24	911 (36.8)	200 (33.8)	
	nulliparous	201 (8.1)	58 (9.8)	
Number of children	0	245 (9.9)	72 (12.1)	0.068
	[1,3[	1694 (68.3)	379 (63.5)	
	$\ge$ 3	541 (21.8)	146 (24.5)	
Total duration of breastfeeding (months)	0	1383 (55.8)	343 (57.6)	0.026
	[1,7[	739 (29.8)	149 (25.0)	
	$\ge$ 7	355 (14.3)	104 (17.4)	
Overweight (BMI $\ge$ 25)	No	2120 (87.7)	488 (85.2)	0.122
	Yes	298 (12.3)	85 (14.8)	
Financial difficulties and/or not going on vacation	No	1518 (61.9)	323 (54.8)	0.002
	Yes	935 (38.1)	266 (45.2)	
Age at menopause (years)	$\ge$ 41	2229 (94.1)	532 (95.3)	0.311
	< 41	139 (5.9)	26 (4.7)	
Mother history of breast cancer	No	2251 (92.4)	517 (87.9)	0.001
	Yes	184 (7.6)	71 (12.1)	
Sister history of breast cancer	No	2261 (91.6)	536 (90.1)	0.268
	Yes	207 (8.4)	59 (9.9)	
Daughter history of breast cancer	No	2451 (99.1)	588 (98.7)	0.499
	Yes	23 (0.9)	8 (1.3)	
Aunt history of breast cancer	No	2271 (93.9)	521 (91.2)	0.029
	Yes	148 (6.1)	50 (8.8)	

We explored whether a selection bias might have occurred. We analyzed the distribution of the two variables (age and breast density) we had for women who could not be reached or who refused to answer our questions. The results are shown in Tables 2 and 3. The only significant difference was observed for breast density of women who did not develop cancer during the follow-up. Women who responded to the survey and did not present a breast cancer during the follow-up period, had a significantly higher breast density on average than women who did not respond to the survey and did not develop a breast cancer. Thus, in women without breast cancer, responders were more at risk than non responders. Under these conservative conditions, we considered that the selection bias would not have penalized the analysis. Moreover, since women are not informed of the degree of their breast density, we did not retain the fact that breast density as such, might have been a criterion to answer the questionnaire, or not.

**Table 2:** Distribution of breast density according to the answer or not to the study questionnaire.

	Breast density	Unreachable or refusal	Answers	P-value
No breast cancer	A	17.2%	13.7%	0.0007
	B	58.1%	59.8%	
	C	21.7%	24.3%	
	D	3.0%	2.2%	
Breast cancer	A	11.7%	10.5%	0.6035
	B	61.4%	58.1%	
	C	23.3%	28.0%	
	D	3.6%	3.4%

**Table 3:** Distribution of age according to the answer or not to the study questionnaire.

	Unreachable or refusal		Answers		P-value
	mean (sd)		mean(sd)		
**No breast cancer**	55	(3.2)	55.1	(3.2)	0.384
**Breast cancer**	55.7	(3.1)	55.3	(3.3)	0.125

### Cox model

**Table 4:** Cox model for the occurrence of breast cancer.

		Hazard Ratio	$95\% CI$	P-value
Age		1.024	0.997-1.05	0.084
Breast density	A	-	-	-
	B	1.411	1.05-1.9	0.023
	C	1.651	1.2-2.27	0.002
	D	2.112	1.25-3.58	0.006
Mother history of breast cancer	No	-	-	-
	Yes	1.612	1.24-2.09	< 0.001
Financial difficulties and/or not going on vacation	No	-	-	-
	Yes	1.299	1.09-1.55	0.003
Age at menopause	$\ge$ 41	-	-	-
	< 41	0.364	0.13-0.99	0.047
Age at menarche	< 12	-	-	-
	$\ge$ 12	0.774	0.63-0.95	0.015
Age at menopause < 41 and Age at menarche $\ge$ 12		2.73	0.91-8.19	0.073

*HR for Age at menopause* < 41 *and* *Age at menarche* $\ge$ *12 =* $0.364 \times 0.774 \times 2.73 = 0.769.$

The final model, presented in Table 4, has been built with 2768 women for whom answers to all the covariates of the model were available, including 2241 non-cancer patients and 527 women who developed cancer during the follow-up period.

The risk factors selected by the model were high breast density (ACR BI-RADS grading) (B vs A: HR = 1.41, 95% CI [1.05; 1.9], p = 0.023; C vs A: HR = 1.65, 95% CI [1.2; 2.27], p = 0.02; D vs A: HR = 2.11, CI [1.25; 3.58],p = 0.006), history of maternal breast cancer (HR = 1.61, 95% CI [1.24; 2.09], p < 0.001), and socioeconomic difficulties (HR = 1.23, 95% CI [1.09; 1.55], p = 0.003). While early menopause (HR = 0.36, 95% CI [0.13; 0.99], p = 0.003) and an age at menarche after 12 years (HR = 0.77, 95% CI [0.63; 0.95], p = 0.047). Age (HR = 1.024, 95% CI [0.997; 1.05], p  =  0.084) and interaction between age at menarche and menopause (HR = 2.73, 95% CI [0.91; 8.19], p  =  0.073) have been forced in the model. The interaction between age at menarche and menopause was forced in the model to take into account the duration of estrogen impregnation, a notable risk factor in our literature searches^28^.

### Bootstrap

We computed 100,000 bootstrap databases using random sampling with replacement among the 2768 women for whom we had answers to all the covariates of the final Cox model. The risk function *F*(*t*) was calculated for all women in the 100,000 databases.

### Threshold determination

Pre-test prevalence of the disease was the percentage of breast cancer in the CRCDC-OC database i.e. 2.6%.

So, as described in Supplementary material 2, we looked for :

- $$s_1$$ such as $$\forall s \ge s_1$$ :
  $$\begin{aligned} LR_+(s)\ge 2.054 \end{aligned}$$
- $$s_2$$ such as $$\forall s \le s_2$$ :
  $$\begin{aligned} LR_-(s)\le 0.493 \end{aligned}$$

**Figure 2:**
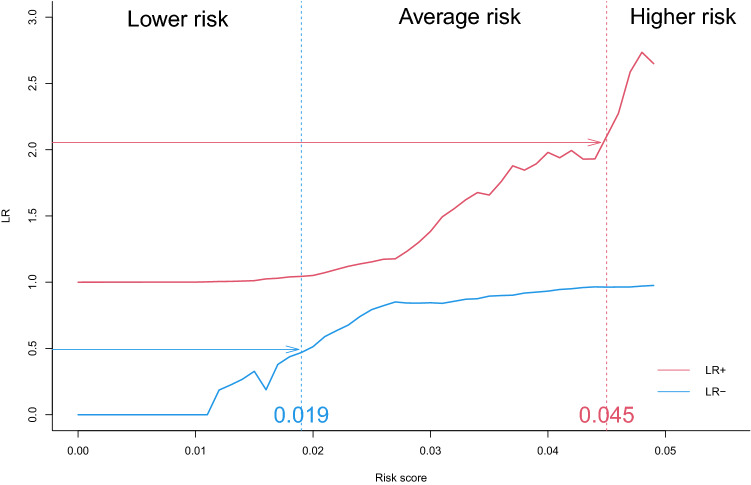
Determination of the three risk groups with optimal thresholds in women at average risk of breast cancer: ”Lower risk”, ”Average risk” and ”Higher risk”.

We then plot LR+(s) and LR-(s) in Fig. 2. We drew the lines $y_1$ and $y_2$ with respective equations $y_1 = 2.054$ and $y_2 = 0.493$. This made it possible to find $s_1$, which is the abscissa of the intersection point between $y_1$ and the representative curve of the $LR_ +$ function, and $s_2$, which is the abscissa of the intersection point between $y_2$ and the representative curve of the $LR_-$ function. This method gave a low threshold at 1.9% and a high threshold at 4.5%.

On one hand, 9.39% of women (469,500 women with a participation rate of 50%) presented a 12-year risk lower than 1.9%. Among this group, the mean 12-year risk was 1.37%. On the other hand, 2.85% of women (145,000 women with a participation rate of 50%) presented a 12-year risk higher than 4.5% with a mean 12-year risk of 5.84%. All this results are presented in Table 5. Of note, if the BC screening participation rate rose to 65%, that would almost represent 800,000 women with a risk different from the ”average” risk.

**Table 5:** Distribution of the organised BC screening population according to the level of risk.

	Mean 12-year risk	Estimated number of targeted women in the organised BC screening population (%)
**Lower risk** (12-year risk < 1.9%)	1.37%	469,500 (9.39%)
**Average risk** (1.9% $\le$ 12-year risk $\le$ 4.5%)	2.6%	4,388,000 (87.76%)
**Higher risk** (12-year risk > 4.5%)	5.84%	142,500 (2.85%)

## Discussion

This work was dedicated to explore whether it was possible to offer a personalized risk based screening to the population of women at « average » risk of BC. In a retrospective cohort of French women, aged 50 to 60, at « average » risk of breast cancer and followed-up for 12 years, from 2006 to 2018, who underwent the BC organized screening, we identified two additional levels of risk, either twice lower than the average-risk, or twice higher. Our partition into three groups at risk of breast cancer in these women showed that the level of risk in the so-called « average » risk population is not homogeneous.

In a first step we built a score of breast cancer risk that included six variables, two of which (age and breast density) were already included in the databases of the French CRCDCs. Only four more questions were required to be asked in order to establish the score. This information is easy and simple to collect at the time of the mammogram.

In a second step, based on the score and likelihood ratios, we identified three levels of risk with a risk ratio of at least 2 between the group at average risk and the group at lower risk on one hand, and on the other hand with the group at higher risk. It lead to fairly large group sizes. Thus, 12.3% of women belonged either to the lower risk group (9.4%) or to the higher risk group (2.9%). We chose to look for a risk divided or multiplied by a factor two. This choice remains arbitrary, though clinically relevant. Moreover, sensitivity analysis could be carried out by varying this ratio according to complementary hypotheses of epidemiologists or physicians specialized in these field. Furthermore, the choice could be supported by a cost function taking into account monetary cost and years of life adjusted for quality of life.

The levels of BC risk are difficult to compare between countries since the clinical guidelines where risk-based screening is recommended differ from country to country, or even in the same country for instance in the United States of America (USA). For the United Kingdom (UK) three levels of risk were delineated by the National Institute for Health and Care Excellence (NICE): near population risk, moderate risk and high risk^29^. The term used by the Nice guidelines of « moderate » BC risk is based on a lifetime risk from age 20 of developing breast cancer of at least 17% but less than 30%. A moderate risk between ages 40 to 50 was estimated between 3% and 8%. The recommendations on surveillance for women at moderate BC risk are modulated according to age. As the likelihood of getting breast cancer increases with age, all women aged from 50 to their 71st birthday who are registered with a GP are automatically invited for breast cancer screening by mammography every 3 years (Guidance NHS Breast screening programme screening standards valid for data collected from 1 April 2021)(www.nhs.uk). The term « average » risk was retained by the National Comprehensive Cancer Network in the US^30^. For the NCCN Panel, increased risk of developing BC is defined by the modified Gail model for women $\ge$35 years of age as a 5-year risk of 1.7% or greater. The United States Preventive Services Task Force (USPSTF) recommends biennial screening mammography for women between the ages of 50 and 74 years^31^. However, the NCCN recommendations are different. For a woman aged 35 years of age or older, with a 5-year risk $\ge$1.7% or greater, defined by the modified Gail model (bcrisktool.cancer.gov) the NCCN Panel encourages breast awareness and recommends a clinical encounter every 6 to 12 months and annual digital mammography, with the consideration of tomosynthesis.

In France, the term “average risk” refers here to the sub-population of women aged 50 to 70 years of age for whom BC screening is indicated, i.e. asymptomatic women who are not at « high risk » . The lifetime risk from age 20 of developing breast cancer in our population was 15.2%. The terms « high risk » or « very high risk » are reserved for women presenting with known risk factors, particularly familial risk factors, or genetic factors such as BRCA-1-2. The Haute Autorité de Santé defined “high” or “very high” risk women^4^. Several contexts justify a specific exploration in women who already experienced a personal history of breast cancer, ductal or in situ lobular carcinoma or atypical lobular or ductal hyperplasia, in women who have had high dose medical chest irradiation (especially for Hodgkin’s disease), and those who experienced some family history of BC. In the event of a BC family history with an Eisinger score equal or greater than 3, which leads to an oncogenetic consultation, AND in the absence of a BRCA 1 or 2 mutation in the family, it is the oncogeneticist that assesses the woman’s personal risk of BC. Women at « high risk » or « very high risk » benefit from individualized monitoring and are not integrated in the French organized BC screening.

Clinical Guideline CG164 for family history of BC were provided by the NICE entitled : classification, care and managing breast cancer and related risks in people with a family history of breast cancer^32^. The NCCN recommendations for Genetic/Familial High-Risk Assessment: Breast, Ovarian, and Pancreatic are also available^33^.

Since we focused our study on women at average risk, we did not include information on genomics or susceptibility genes in our questionnaire. However, several studies^12,34,35^ targeted breast cancer susceptibility genes in order to better personalize the screening procedure. Moreover, two ongoing studies explore risk based screening strategies that include genomic tests and polygenic risk scores. The “WISDOM” study (Women Informed to Screen Depending On Measures of risk) is a multicenter trial comparing risk-based screening to annual screening in 100,000 women aged 40-74, initially launched in the Athena Breast Health Network in California and the Midwest^36^. It is based on personalized breast cancer screening recommendations based on individual risk assessments. Components of individual risk assessment are based on integral biomarkers: Breast Cancer Surveillance Consortium 5-year risk; genomic tests for rare high/moderate-penetrance mutations in a number of genes, including the following: BRCA1, BRCA2, ATM, CDH1, CHEK2, PALB2, PTEN, STK11 and TP53; and polygenic risk score from 96 lower-risk common genetic variants (SNPs) with known association to breast cancer, updated as data emerge. Two primary endpoints were defined: safety is explored through a non-inferiority hypothesis comparing the rate of stage IIB cancers or higher diagnosed in annual versus risk-based screening arms. Morbidity evaluation is based on a reduced rate of recall and breast biopsy between arms. Another ongoing study is « MyPebs » (My Personal Breast Screening)^37^ which is an international randomized, open-label, multicentric study assessing the effectiveness of a risk-based breast cancer screening strategy, which uses clinical risk scores and polymorphisms, compared to standard screening, according to the current national guidelines in each participating country, in detecting stage II or higher breast cancers. Women aged 40 to 70 years old are differentially screened for 4 years^38^.

The recruitment of WISDOM or MyPebs studies differ from our’s. In our study, we included women aged 50 to 62 years, and followed-up for 12 years. Moreover, the French screening is not based on annual screening but on biennial mammogram. Moreover, unlike these two studies, we took into account all types of breast cancer and not just stage II or higher cancers.

In our study we did not include genomic tests, neither search for genetic variants. In our sample, only 1.5% of women of the lower risk group had a mother who presented a BC. It was 6.1% in the average group. Conversely, it was 71.7% in the higher risk group. We considered that genomic tests including 9 genes and 96 to 258 SNPs in women at average risk is not currently a screening approach that can be extended in France to 10 million women aged between 50 and 74 years. Moreover, the costs efficiency of such a strategy, financial as well as organizational, has not been evaluated yet. It is also worth noting that the penetrance of a particular pathogenic germline variant (PGV) in a patient without a pretest probability that satisfies the National Comprehensive Cancer Network recommendations for targeted testing for that PGV, is unclear for most PGVs^33^. In effect, it remains difficult to estimate penetrance when a particular PGV is discovered in a woman who lacks a significant pretest probability of carrying this particular PGV^39–41^.

Women interviewed during the French citizen consultation on breast cancer screening in 2017, expected the organized screening to be more targeted^42^, thanks to advances in research, and to become more personalized according to individual risk factors. Physicians, also consulted, mentioned the value of determining the level of individual risk^7,43^. They also ask for a score in French women in order to substantiate the levels of risk. In this perspective, we developed a user friendly computer application, mainly dedicated to physicians, that enables estimating the value of BC risk for a given woman, and provides an aid to personalizing the organized screening accordingly (application available at shiny.temas-bonnet.site/breast_cancer).

In France, the organized BC screening enables a regular follow-up of invited women, aged 50 to 74, to get a biennial mammogram, covered by health insurance, in the radiology office of their choice. Moreover, even if the initial reading of the mammogram does not present any anomaly, a second reading is systematically performed by another radiologist. The second independent reading is a guarantee of reliability, with almost 8% of the breast cancers detected by the independent second reading^44,45^.

The principle of an organized breast cancer screening has been challenged given the claims of over-diagnosis or over-treatment (references). A reassessment of the organized screening that would allow a personalization according to the individual level of risk, would enable a standardized and quality monitoring of women, while producing evaluative data.

Depending on the presence or level of clinical, genetic or biological markers, treatment strategies are personalized to be as effective as possible. This approach can be extended to public health and in particular for primary and secondary prevention. Indeed, offering personalized prevention strategies could increase adherence to the the screening program, and effectiveness as well. Thus, identifying new groups at risk among women at average BC risk offers new perspectives. According to the two highlighted thresholds of lower and higher risk, 12% of women were thus identified, corresponding to more than 600,000 women in the French population. In this population at ”average” risk of breast cancer, a new screening strategy might therefore be proposed for the groups at lower or higher risk. For the group at average-risk of breast cancer, corresponding to 88% of the presently screened population, the current screening rate, i.e. a mammogram every two years, would be maintained as such.

For the group at lower risk that includes 9.4% of the screened population with a level of risk of 1.37% of developing breast cancer within the 12 years of follow-up, we propose to reduce the frequency of mammograms with a mammogram every three or more years instead of the two years currently in force. This mammogram spacing could reduce the risk of over-diagnosis while maintaining a regular follow-up. Moreover, it would reduce, the risk of radiation-induced cancer, even if it is already very low. However, the risk of interval cancer can’t be excluded. This proposal will have thus to be tested with an appropriate design.

For 2.9% of women at higher risk that we identified among women of our BC screening population, if they fulfill the criteria above defined by the Haute Autorité de Santé^4^ an oncogenetic consultation is required. Otherwise, particularly in the group of high breast density, increasing the frequency of mammograms is conceivable. However, high breast density is an important risk factor for this group where the distribution of high or very high breast density was 60% compared to 28% in the group at average risk and 3% in the group at lower risk. Furthermore, the mammogram sensitivity falls to 63%^46^ for very dense breasts. The sensitivity of cancer detection via a mammogram appeared inversely proportional to breast density^47^. Thus, it did not seem that increasing the frequency of mammograms would bring a significant benefit. Based on recent results, an alternative would be to offer these women an abbreviated breast MRI, which appeared associated with a higher rate of breast cancer detection among women with dense breasts^48^, while maintaining a two-year frequency for surveillance.

Such a personalization of the organized screening might make women feeling more involved. For women at lower risk, a mammogram every three years would be less restrictive than every two years. For women at higher risk (excluding women for whom an oncogenetic consultation is indicated), offering an intensified surveillance may increase their adhesion to an adapted screening. This strategy might contribute to increase the participation of French women to the organized screening which is currently around 50%^5^, although the target is 65%.

As previously stated, the case finding costs (including diagnosis and treatment costs) should be economically balanced in connection with possible expenditure on medical care as a whole^49^. Analysis of the medico-economic gain should be carried out taking into account, among other things, interval cancers, over-diagnosis and the stage of detected BC. In this observational survey, the difficulty to contact women is inherent in this type of design and might introduce a selection bias. In our study, among non-participating women, 4 out of 5 were not reachable by phone, however it did not introduce a selection bias since it is at random. Conversely, 1 in 5 women indeed refused to answer the questionnaire which might have introduced a selection bias, even if the majority of them alleged a lack of time to fill out the questionnaire. We considered that it was not obviously linked to the level of BC risk, though limiting a potential selection bias.

An internal validation on another database, for example of women aged 40 to 50 in the Hérault department, would be of interest. External geographic validation might be carried out on the databases from other CDCRCs and/or a database from another country^50^. Moreover, information of interest for the creation of our score was collected at $t_0$, the inclusion time. But, over time, the value of variables retained at $t_0$ may change and the risk at $t_0 + x$ years may also change. It will therefore be necessary to regularly re-estimate risk of BC in order to assign women to the appropriate group of risk. A re-evaluation of the score will also be necessary on regular time intervals to reappraise the coefficients estimated by the model in order to evaluate whether they would need to be adjusted. In this study, we proposed a new approach to personalize the organized screening by stratifying the risk within the population at ”average” risk and currently considered as homogeneous. However, the results of this study remain to be confirmed in other contexts.

## Supplementary Information

Supplementary Information 1.

Supplementary Information 2.

Supplementary Information 3.

## Data Availability

The data used and analyzed during the current study are available from the corresponding author (EB).

## Code availability

The code used during the current study is available from the corresponding author (EB).
