# Supplementary material for: Determination of thresholds of risk in women at average risk of breast cancer to personalize the organized screening program
Source: Sci Rep. 2021 Sep 27;11:19104. doi: 10.1038/s41598-021-98604-6 (PMC8476568; doi:10.1038/s41598-021-98604-6)
Supplement: Supplementary file 1 — Supplementary Information 1. [file 41598_2021_98604_MOESM1_ESM.pdf]

Supplementary material 1

Determination of thresholds of risk in women at average risk of breast cancer to personalize the organized screening program.

Bonnet E, Daures JP, Landais P

Questionnaire on Breast Cancer Risk Factors in French Women.

<u>We'll start by asking you a few questions about your personal background:</u>

1) Have you ever had breast cancer?
   ☐ Yes
   ☐ No

2) How old were you at your first period? |_|_| years

3) Have you ever used birth control pills **before 2006**?
   ☐ Yes ➔ If so, for how long? |_|_| years
   ☐ No

4) Have you had any pregnancies **before 2006**:

   ☐ Yes ➔ If so :
   - Number of pregnancies? |_|_|
   - Age at your first pregnancy: |_|_| years

   ☐ No

5) How many children have you had **before 2006** ? |_|_|
   - How many girls among these children? |_|_|

6) What was your total duration of breastfeeding **before 2006**? (If you breastfed more than one child this includes the **cumulative breastfeeding time of all your children**) |_|_| monthes

7) Do you have sisters?
   ☐ Yes ➔ If yes, how many? |_|_|
   ☐ No

8) Were you postmenopausal **in 2006**?
   ☐ Yes ➔ If yes, age at menopause? |_|_| years
   ☐ No
   ☐ Do not know

9) Have you taken hormonal therapy for menopause **before 2006**? (**if you were postmenopausal in 2006**)

☐ No
☐ Yes: If yes,

- Number of years of treatment **before 2006** ? |_|_| years.
- What treatment did you take?

↳ __________________ ☐ Do not know

10) Have you had breast biopsies **before 2006**?

☐ No
☐ Yes ➔ If yes, how many ? |_|_|

Result of breast biopsies?

☐ Normal
☐ Non-proliferative lesion
☐ Proliferation without atypia
☐ Proliferation with atypia
☐ Lobular carcinoma in situ
☐ Unknown

11) Have you had any treatment for osteoporosis **before 2006** ?

☐ Do not know
☐ No
☐ Yes ➔ If yes, which ones? (if several check several boxes)

Raloxifène (Evista, Optruma) ☐ Yes ☐ No ☐ Do not know
Biphosphonates (Didronel, Skelid, Alendronate, Actonel, Clastoban, Fosamax, Bonviva) ☐ Yes ☐ No ☐ Do not know
Tériparatide (Forsteo) ☐ Yes ☐ No ☐ Do not know
Dénosumab (Prolia, Xgeva) ☐ Yes ☐ No ☐ Do not know
Other : ☐ Yes ☐ No, If yes, name ?__________________________

12) Have you had any fractures not caused by major trauma **before 2006**?

☐ No
☐ Yes ➔ If yes, where?

Wrist : ☐ Yes ☐ No
Femur : ☐ Yes ☐ No
Compressed vertebra : ☐ Yes ☐ No

13) At the age of 40, what was your :

- height |_|_|_|cm
- average weight |_|_|_|Kg

14) After menopause what was your : (**if menopaused in 2006**)

- average weight |_|_|_|Kg

15) Have you had cancer **before 2006** ? (other than breast cancer)

☐ No

☐ Yes ➔ If yes, where ? (if several check several boxes)
☐ Ovary
☐ Colon
☐ Lung
➔ If yes, have you received any treatment by radiotherapy? ☐ Yes / ☐ No
☐ Other location ➔ If yes, where?______________________

16) Have you had Hodgkin's disease?
☐ No
☐ Yes ➔ If yes, have you had chest radiation therapy?
☐ Yes
☐ No

We will now ask you about your lifestyle and socio-economic habits:

17) What was your physical activity **before 2006**:
- Number of hours of sport per week: |_|_| hours/week
- For how many years **before 2006**? |_|_| years

18) **In 2006,** per week, how much did you drink:
- Glasses of wine? (10cl) |_|_|
- Can / bottle of beer? (25cl) |_|_|
- Dose of strong alcohol (3cl of 40 ° alcohol)|_|_|

- For how many years **before 2006**? |_|_| years

19) Were there periods in the month when you encountered real financial difficulties in meeting your needs (food, rent, electricity bill, etc.) **before 2006**?
☐ Yes
☐ No

20) Did you go on vacation regularly **before 2006**?
☐ Yes
☐ No

21) In case of difficulties, were there people around you who you could count on to accommodate you for a few days in needed **before 2006**?
☐ Yes
☐ No

22) In case of difficulties, were there people around you who you could count on to provide you with material or financial assistance **before 2006**?
☐ Yes
☐ No

We will end with your family history of cancer:

23) About your family history of **breast cancer** :

	Yes	No	Do not know	Non pertinent	**If Yes** At what age did the person develop this cancer?	**If Yes** In which year?
Mother with breast cancer						
1st sister with breast cancer						
2nd sister with breast cancer						
3rd sister with breast cancer						
1st daughter with breast cancer						
2nd daughter with breast cancer						
3rd daughter with breast cancer						
1st maternal aunt with breast cancer						
2nd maternal aunt with breast cancer						
3rd maternal aunt with breast cancer						
Father with breast cancer						
1st paternal aunt with breast cancer						
2nd paternal aunt with breast cancer						
3rd paternal aunt with breast cancer						

24) About your family history of **ovarian cancer** :

	Yes	No	Do not know	Non pertinent	**If Yes** At what age did the person develop this cancer?	In which year?
Mother with ovarian cancer						
1st sister with ovarian cancer						
2nd sister with ovarian cancer						
3rd sister with ovarian cancer						
1st daughter with ovarian cancer						
2nd daughter with ovarian cancer						
3rd daughter with ovarian cancer						
1st maternal aunt with ovarian cancer						
2nd maternal aunt with ovarian cancer						
3rd maternal aunt with ovarian cancer						
1st paternal aunt with ovarian cancer						
2nd paternal aunt with ovarian cancer						
3rd paternal aunt with ovarian cancer						

Thank you for your cooperation, The Clinical Research Team.
