# Supplementary material for: Determination of thresholds of risk in women at average risk of breast cancer to personalize the organized screening program
Source: Sci Rep. 2021 Sep 27;11:19104. doi: 10.1038/s41598-021-98604-6 (PMC8476568; doi:10.1038/s41598-021-98604-6)
Supplement: Supplementary file 2 — Supplementary Information 2. [file 41598_2021_98604_MOESM2_ESM.pdf]

## Supplementary material 2

**Determination of thresholds of risk in women at average risk of breast cancer to personalize the organized screening program.**

Bonnet E, Daures JP, Landais P

# Thresholds determination

We want:

$$\frac{\mathbb{P}(M|T > s)}{\mathbb{P}(M|T < s)} \geq 2 \qquad \forall s \geq s_1$$

and

$$\frac{\mathbb{P}(M|T < s)}{\mathbb{P}(M|T > s)} \leq 0.5 \qquad \forall s \leq s_2$$

For the higher threshold $\mathbb{P}(M|T < s)\#\mathbb{P}(M)$. So, in this first case we want: $\forall s \geq s_1$:

$$\frac{\mathbb{P}(M|T > s)}{\mathbb{P}(M)} \geq 2$$

Moreover :

$$\begin{aligned}
\mathbb{P}(M|T > s) &= \frac{\mathbb{P}(M \cap T > s)}{\mathbb{P}(T > s)} \\
&= \frac{\mathbb{P}(T > s|M)\mathbb{P}(M)}{\mathbb{P}(T > s)} \\
&= \frac{\mathbb{P}(T > s|M)\mathbb{P}(M)}{\mathbb{P}(T > s|M)\mathbb{P}(M) + \mathbb{P}(T > s|\bar{M})\mathbb{P}(\bar{M})} \\
&= \frac{\text{Se}(s)\mathbb{P}(M)}{\text{Se}(s)\mathbb{P}(M) + (1 - \text{Sp}(s))(1 - \mathbb{P}(M))}
\end{aligned}$$

So,

$$\begin{aligned}
\frac{\mathbb{P}(M|T>s)}{\mathbb{P}(M)} &= \frac{\text{Se}(s)}{\text{Se}(s)\mathbb{P}(M)+(1-\text{Sp}(s))(1-\mathbb{P}(M))} \\
&= \frac{\text{Se}(s)}{\mathbb{P}(M)(\text{Se}(s)+\text{Sp}(s)-1)+(1-\text{Sp}(s))} \\
&= \frac{\frac{\text{Se}(s)}{1-\text{Sp}(s)}}{\mathbb{P}(M)\left(\frac{\text{Se}(s)}{1-\text{Sp}(s)}-1\right)+1} \\
&= \frac{LR_+(s)}{\mathbb{P}(M)(LR_+(s)-1)+1}
\end{aligned}$$

Thus,

$$\begin{aligned}
& \frac{\mathbb{P}(M|T>s)}{\mathbb{P}(M)} \geq 2 \\
\Rightarrow\ & \frac{LR_+(s)}{\mathbb{P}(M)(LR_+(s)-1)+1} \geq 2 \\
\Rightarrow\ & LR_+(s) \geq 2\left[\mathbb{P}(M)(LR_+(s)-1)+1\right] \text{ with } LR_+ \text{ increasing} \\
\Rightarrow\ & LR_+(s) \geq 2\left[\mathbb{P}(M)LR_+(s)-\mathbb{P}(M)+1\right] \\
\Rightarrow\ & LR_+(s) \geq 2\mathbb{P}(M)LR_+(s)+2(1-\mathbb{P}(M)) \\
\Rightarrow\ & LR_+(s)-2\mathbb{P}(M)LR_+(s) \geq 2(1-\mathbb{P}(M)) \\
\Rightarrow\ & LR_+(s)\left[1-2\mathbb{P}(M)\right] \geq 2(1-\mathbb{P}(M)) \\
\Rightarrow\ & LR_+(s) \geq 2\frac{1-\mathbb{P}(M)}{1-2\mathbb{P}(M)}
\end{aligned}$$

With $\mathbb{P}(M) = 2.6\%$, since $LR_+$ is increasing, we look for $s_1$ as $\forall s \geq s_1$ :

$$LR_+(s) \geq LR_+(s_1) = 2.054$$

For the lower threshold, $\mathbb{P}(M|T>s)\#\mathbb{P}(M)$. In this second case we want that, $\forall s \geq s_1$:

$$\frac{\mathbb{P}(M|T<s)}{\mathbb{P}(M)} \leq 0.5$$

With,

$$\begin{aligned}
\mathbb{P}(M|T<s) &= \frac{\mathbb{P}(T<s|M)\mathbb{P}(M)}{\mathbb{P}(T<s)} \\
&= \frac{\mathbb{P}(T<s|M)\mathbb{P}(M)}{\mathbb{P}(T<s|M)\mathbb{P}(M)+\mathbb{P}(T<s|\bar{M})\mathbb{P}(\bar{M})} \\
&= \frac{(1-\text{Se}(s))\mathbb{P}(M)}{(1-\text{Se}(s))\mathbb{P}(M)+\text{Sp}(s)(1-\mathbb{P}(M))}
\end{aligned}$$

So,

$$\begin{aligned}
\frac{\mathbb{P}(M|T < s)}{\mathbb{P}(M)} &= \frac{(1-\text{Se}(s))}{(1-\text{Se}(s))\mathbb{P}(M) + \text{Sp}(s)(1-\mathbb{P}(M))} \\
&= \frac{\frac{1-\text{Se}(s)}{\text{Sp}(s)}}{\mathbb{P}(M)\frac{1-\text{Se}(s)}{\text{Sp}(s)} + (1-\mathbb{P}(M))} \\
&= \frac{LR_-(s)}{\mathbb{P}(M)LR_-(s) + (1-\mathbb{P}(M))}
\end{aligned}$$

Thus,

$$\begin{aligned}
& \frac{\mathbb{P}(M|T < s)}{\mathbb{P}(M)} \leq 0.5 \\
\Rightarrow\ & \frac{LR_-(s)}{\mathbb{P}(M)LR_-(s) + (1-\mathbb{P}(M))} \leq 0.5 \\
\Rightarrow\ & LR_-(s) \leq 0.5\left[\mathbb{P}(M)LR_-(s) + (1-\mathbb{P}(M))\right] \quad \text{with } LR_- \text{ increasing} \\
\Rightarrow\ & LR_-(s) \leq 0.5\mathbb{P}(M)LR_-(s) + 0.5(1-\mathbb{P}(M)) \\
\Rightarrow\ & LR_-(s) - 0.5\mathbb{P}(M)LR_-(s) \leq 0.5(1-\mathbb{P}(M)) \\
\Rightarrow\ & LR_-(s)\left[1 - 0.5\mathbb{P}(M)\right] \leq 0.5(1-\mathbb{P}(M)) \\
\Rightarrow\ & LR_-(s) \leq 0.5\frac{1-\mathbb{P}(M)}{1-0.5\mathbb{P}(M)}
\end{aligned}$$

With $\mathbb{P}(M) = 2.6\%$, since $LR_-$ is increasing, we look for $s_2$ as $\forall s \leq s_2$ :

$$LR_-(s) \leq LR_-(s_1) = 0.493$$
