# Supplementary material for: Determination of thresholds of risk in women at average risk of breast cancer to personalize the organized screening program
Source: Sci Rep. 2021 Sep 27;11:19104. doi: 10.1038/s41598-021-98604-6 (PMC8476568; doi:10.1038/s41598-021-98604-6)
Supplement: Supplementary file 3 — Supplementary Information 3. [file 41598_2021_98604_MOESM3_ESM.pdf]

Supplementary material 3

Determination of thresholds of risk in women at average risk of breast cancer to personalize the organized screening program.

Bonnet E, Daurès JP, Landais P

INFORMATION LETTER

Determination of thresholds of risk in women at average-risk of breast cancer in order to personalize the organized screening program. DOPEKS Study.

ID-RCB : 2020-A02460-39

Coordinating investigator: Pr Paul LANDAIS
EA 2415
AiDMP – Aide à la Décision pour une Médecine Personnalisée
Address: 75 Rue des Professeurs Truc, 34090 Montpellier
TEL 04 11 75 98 41

Promoter:
Clinique Beau Soleil
119 avenue de Lodève
34000 Montpellier
Tel : 04.67.75.98.47

Madam,

You are asked to participate in an observational study promoted by the Beau Soleil clinic. The purpose of this document is to provide you with the information necessary for your decision. You can discuss this with someone you trust or your doctor. Our investigating doctor is also at your disposal to answer all your questions and explain to you what you do not understand.

You are free to accept or refuse to participate in this research, and you can be accompanied by a person you trust.

What is the aim of this study?

You were screened by a mammography examination between 2006 and 2007, whatever the result of this examination, we would like to supplement your medical data with a questionnaire in order to build a score aiming to identify women at risk of breast cancer. This score might make it possible to better adapt the monitoring of breast cancer screening according to the results of this study.

What does this study consist of?

This study consists of responding to a questionnaire concerning your family and personal history, your physical activity and your lifestyle and socio-economic habits. This data is collected during a telephone interview of approximately 15 minutes.

During this interview, data is collected by trained medical investigator respecting medical confidentiality. All recorded data is anonymized. Thus, no personal data which would allow you to be identified is collected.

What is the schedule for this study?

For you, the study lasts the time of the telephone interview. You will find attached the questions that will be asked.

How many people will participate in this study?

It is planned to include 3765 women, drawn at random from the women who underwent organized breast cancer screening in 2006 or 2007. Aged 50 to 60 in 2006-2007 and who did not develop breast cancer before that date.

What are the possible benefits and risks of the study?

There is no direct benefit to you, but the results may benefit future generations. Likewise, no risks or constraints for you are expected: carrying out your screening and your care does not differ from the usual procedure.

The results of the study may be communicated to you at the end of the research.

What are your rights?

As part of this research in which the Beau Soleil clinic invites you to participate, processing of your personal data will be implemented to allow the analysis of the results. For this purpose, the collected medical data is transmitted to us. These data are identified by a code number which does not identify you.

This study complies with the “Reference Method MR-003” established by the Commission Nationale Informatique et Liberté (CNIL). The Beau Soleil clinic, as the study sponsor, has signed a commitment to comply with this "Reference Methodology" which guarantees that the processing of personal data follows the requirements of the CNIL.

The staff involved in the study are subject to medical confidentiality, as is your general practitioner.

In accordance with the provisions of the law relating to computers, files, and freedoms (law of January 6, 1978 amended) and European regulation 2016/679 of April 27, 2016, you have a right of access, portability, rectification, erasure and limitation of your personal data.

The Beau Soleil clinic is the data controller within the meaning of European Regulation 2016/679, since it determines the purposes and means of data processing as part of this study. Article 9 of this regulation allows it to process special categories of data, including health data.

You also have the right to oppose the transmission of data covered by medical confidentiality that may be used in the context of this research and that may be processed. The exercise of this right entails the termination of participation in the study. Your data will be kept for a period of 15 years from the end of the study.

You can also access all of your medical data directly or through a doctor of your choice in accordance with the provisions of Article L 1111-7 of the Public Health Code. These rights are exercised with the doctor who follows you in the context of the research and who knows your identity or with the Protection Officer

of the Beau Soleil clinic. For any complaint relating to the processing of your health data, you can contact the CNIL.

In accordance with law n ° 2004-806 of August 9, 2004 relating to public health policy, the Committee for the **Protection of Persons of Nice** studied this research project and issued a **favorable** opinion for its realization.

Your non-opposition to this research must be informed and voluntary. Your participation is optional and your refusal will in not affect the quality of care and your relationship with the medical team.

The investigator: Pr Paul Landais

Signature :
